# Calendar

**DOI:** 10.1111/iwj.13881

**Published:** 2022-07-15

**NORTH AMERICA**

**28‐31 July 2022**

***American Podiatric Medical Association (APMA) Annual Scientific Meeting***

**Orlando, Florida, USA**

www.apma.org/events/thenational.cfm

**17‐20 August 2022**

***Annual Amputation Prevention Symposium (AMP*** ***)***

**Chicago, Illinois, USA**

www.amptheclimeeting.com/amp‐2022

**26‐28 August 2022**

***Modern Wound Care Management***

**Stevenson, Wyoming USA**

www. modernwound.com

**29 September – 1 October 2022**

***SVM 33rd Annual Scientific Sessions***

**Grand Hyatt Denver, Denver, Colorado USA**

www.woundsource.com/resource/svm‐33rd‐annual‐scientific‐sessions

**EUROPE**

**19‐20 July 2022**

***International Conference on Novel Biomaterials in Drug Delivery and Wound Care ICNBDDWC***

**Copenhagen, Denmark**

www.waset.org/novel-biomaterials-in-drug-delivery-and-wound-care-conference-in-july-2022-in-copenhagen

**15‐18 August 2022**

***International Surgical Week 2022 (ISW 2022*** ***)***

**Vienna, Austria**

www.isw2022.org

**7‐10 September 2022**

***International Continence Society Annual Meeting***

**Vienna, Austria**

www.woundsource.com/resource/international‐continence‐society‐annual‐meeting

**7–10 September 2022**

***31st EADV Congress***

**Milan, Italy & ONLINE**

www.eadvcongress2022.org

**14–16 September 2022**

***EPUAP 2022 (22nd Annual Meeting of the European Pressure Ulcer Advisory Panel*** ***)***

**Prague, Czech Republic**

www.epuap2022.org

**14–17 September 2022**

***Wounds Australia National Conference 2022***

**Sydney, Australia**

www.woundsaustralia.com.au/Web/Events/Web/Events/Event_List.aspx

**16–18 September 2022**

***18th Meeting of the Diabetic Foot Study Group (DFSG*** **)**

**Bratislava, Slovakia**

www.dfsg.org/1/dfsg‐2022

